# Gastrostomy in patients with amyotrophic lateral sclerosis (ProGas): a prospective cohort study

**DOI:** 10.1016/S1474-4422(15)00104-0

**Published:** 2015-07

**Authors:** ProGas Study Group

## Abstract

**Background:**

Gastrostomy feeding is commonly used to support patients with amyotrophic lateral sclerosis who develop severe dysphagia. Although recommended by both the American Academy of Neurology and the European Federation of Neurological Societies, currently little evidence indicates the optimum method and timing for gastrostomy insertion. We aimed to compare gastrostomy insertion approaches in terms of safety and clinical outcomes.

**Methods:**

In this large, longitudinal, prospective cohort study (ProGas), we enrolled patients with a diagnosis of definite, probable, laboratory supported, or possible amyotrophic lateral sclerosis who had agreed with their treating clinicians to undergo gastrostomy at 24 motor neuron disease care centres or clinics in the UK. The primary outcome was 30-day mortality after gastrostomy. This study was registered on the UK Clinical Research Network database, identification number 9923.

**Findings:**

Between Nov 2, 2010, and Jan 31, 2014, 345 patients were recruited of whom 330 had gastrostomy. 163 (49%) patients underwent percutaneous endoscopic gastrostomy, 121 (37%) underwent radiologically inserted gastrostomy, 43 (13%) underwent per-oral image-guided gastrostomy, and three (1%) underwent surgical gastrostomy. 12 patients (4%, 95% CI 2·1–6·2) died within the first 30 days after gastrostomy: five (3%) of 163 after percutaneous endoscopic gastrostomy, four (3%) of 121 after radiologically inserted gastrostomy, and three (7%) of 43 after per-oral image-guided gastrostomy (p=0·46). Including repeat attempts in 14 patients, 21 (6%) of 344 gastrostomy procedures could not be completed: 11 (6%) of 171 percutaneous endoscopic gastrostomies, seven (6%) of 121 radiologically inserted gastrostomies, and three (6%) of 45 per-oral image-guided gastrostomies (p=0·947).

**Interpretation:**

The three methods of gastrostomy seemed to be as safe as each other in relation to survival and procedural complications. In the absence of data from randomised trials, our findings could inform clinicians and patients in reaching decisions about gastrostomy and will stimulate further research into the nutritional management in patients with amyotrophic lateral sclerosis.

**Funding:**

Motor Neurone Disease Association of Great Britain and Northern Ireland (MNDA) and the Sheffield Institute for Translational Neuroscience (SITraN).

## Introduction

Amyotrophic lateral sclerosis is a neurodegenerative illness causing progressive weakness and wasting of muscles controlling movement, breathing, and swallowing.^1^ Dysphagia is a common problem in patients with amyotrophic lateral sclerosis and causes difficulties in maintaining a safe and adequate oral intake of nutrition and fluids.^2^ Patients with severe dysphagia often experience weight loss, choking, and coughing on attempting to swallow, episodes of aspiration, and prolonged and effortful mealtimes.^3–6^

Gastrostomy feeding is recommended to provide long-term nutritional support for patients with amyotrophic lateral sclerosis with severe dysphagia.^7^ Three main methods of gastrostomy insertion are currently used in patients with amyotrophic lateral sclerosis: percutaneous endoscopic gastrostomy, radiologically inserted gastrostomy, and per-oral image-guided gastrostomy.^8^ However, with little evidence available,^9,10^ current practice in relation to choice of method and timing of gastrostomy insertion is largely based on consensus and expert opinion.^8^ Gastrostomy could be beneficial for the survival, quality of life, and nutritional outcome of patients with this disease, but there is a paucity of high-quality evidence relating to these aspects of the intervention.^9,11–13^

In response to the paucity of evidence and calls by organisations such as the American Academy of Neurology and the European Federation of Neurological Societies for more evidence to guide clinicians and optimise standards of care,^7,14^ we aimed to identify the optimum gastrostomy timing and insertion method in terms of safety and clinical outcomes.

## Methods

### Study design and participants

In this large, multicentre, longitudinal, prospective cohort study (ProGas), we enrolled patients with a diagnosis of definite, probable, laboratory supported, or possible amyotrophic lateral sclerosis (as defined by the El Escorial criteria),^15^ who had agreed with their clinicians to undergo gastrostomy at one of 24 motor neuron disease care centres or clinics in the UK (21 in England, two in Scotland, and one in Northern Ireland). Patients who had been diagnosed with a disorder characterised by cognitive impairment, such as frontotemporal dementia, were excluded. Patients were approached and invited to take part in the study by a member of the research team when a decision had been made to refer the patient for a gastrostomy insertion. Ethical approval was granted by the National Health Service NRES Leeds (Central) Research Ethics Committee and applied to all participating care centres or clinics. Informal carers, such as family members, of patients who had accepted to take part in the study were also invited to participate. All participants who agreed to take part in the study provided written informed consent before data collection.

**Research in context**

**Evidence before this study**

We searched PubMed, Embase, The Cochrane Library, and ISI Web of Knowledge for reports published before July 1, 2010, combined with citation searching and reference chaining, using the keywords: “motor neuron* disease” or “MND”, “amyotrophic lateral sclerosis” or “ALS”, “gastrostomy”, “percutaneous endoscopic gastrostomy” or “PEG”, “radiologically-inserted gastrostomy” or “RIG”, “per-oral image-guided gastrostomy” or “PIG”, “timing”, “mortality”, “safety”, “nutritional outcome”, “benefits”, and “quality of life”. We identified several studies reporting mortality data after gastrostomy insertion in patients with amyotrophic lateral sclerosis, but only a handful directly compared survival time or 30-day post-procedure mortality after different methods of gastrostomy. In a meta-analysis of the data of the four studies that allowed within-study comparisons of percutaneous endoscopic gastrostomy versus radiologically inserted gastrostomy or per-oral image-guided gastrostomy, the difference in 30-day mortality was increased by 2·1% for percutaneous endoscopic gastrostomy compared with the other insertion methods. However, the results of the meta-analysis did not provide robust evidence to indicate which method is safer because of an absence of within-study comparisons, differences between populations, small sample sizes, and low event rates. The urgent need for prospective clinical trials in relation to the optimum method and timing for gastrostomy insertion, as well as the nutritional outcome for the patients, was highlighted in a Cochrane review on enteral tube feeding for amyotrophic lateral sclerosis and echoed in calls for more robust evidence by multiple organisations, such as the American Academy of Neurology and the European Federation of Neurological Societies.

**Added value of this study**

To our knowledge, ProGas is the first large, multicentre, longitudinal, cohort study to assess and compare the different methods of gastrostomy and explore the issue of optimal timing for gastrostomy insertion in patients with amyotrophic lateral sclerosis.

**Implications of all the available evidence**

In the absence of data from randomised trials, our findings might help neurologists, patients with amyotrophic lateral sclerosis, and the carers of patients with amyotrophic sclerosis to make decisions about the timing and method of gastrostomy. The next steps in building the evidence base must be to understand further the nutritional requirements of patients with amyotrophic lateral sclerosis, particularly the quantity and quality of nutritional support that patients receive after gastrostomy, and to explore the factors that can lead to continuing weight loss after the procedure.

### Procedures

Adhering to the study protocol and the National Institute for Health Research guidelines for good clinical practice, data collection was carried out by experienced members of the local research teams. Data were collected at four timepoints: at the time of recruitment (baseline), at the end of the gastrostomy procedure, at 3 months after gastrostomy, and at 12 months after gastrostomy. At baseline, we collected the following information: demographic characteristics; clinician opinion on indication, timing, potential benefits, and preferred type of gastrostomy; patient's influence on the timing of gastrostomy; measures of respiratory function; and indices of disease progression. At baseline, 3 months, and 12 months we collected the following information: demographic characteristics, weight, height, and score on the revised amyotrophic lateral sclerosis functional rating scale (ALSFRS-R).^16^ Data related to the operation itself such as gastrostomy equipment, type of gastrostomy tube used, procedure length, and details of any complications were collected at the end of the gastrostomy procedure. At baseline and 3 months after gastrostomy insertion, patients who gave consent were asked to complete a questionnaire assessing quality of life (MQOL)^17^ and a questionnaire assessing the strain of caregiving activities was completed by consenting informal carers (MCSI).^18^

### Outcomes

The primary outcome of the study was 30-day mortality after gastrostomy.^8^ The secondary outcomes were perigastrostomy and post-gastrostomy complication rate (defined as complications that occurred during the gastrostomy procedure and those that occurred at any timepoint in the first 3 months after completion of the gastrostomy insertion procedure, respectively), median survival time from gastrostomy placement, nutritional status change, self-perceived quality of life changes after gastrostomy, and changes in carer strain after gastrostomy.

### Statistical analysis

Assuming a 30-day mortality rate of 5%, based on a meta-analysis of the available literature,^8^ to estimate mortality within greater or less than 2·5% (ie, 95% CI 2·5–7·5) would require 30-day mortality data for a minimum of 300 patients with amyotrophic lateral sclerosis. Current European Federation of Neurological Societies guidelines recommend gastrostomy after weight loss of at least 10% from premorbid weight.^14^ This threshold was used in our study to classify patients into weight loss subgroups for subsequent analyses. Continuity corrected χ^2^ tests were done to determine the difference in the 30-day mortality and the complication rates after gastrostomy in patients who underwent percutaneous endoscopic gastrostomy, radiologically inserted gastrostomy, or per-oral image-guided gastrostomy. Kaplan-Meier survival curves were used to determine the median survival time from placement and disease onset for the treatment groups. Cox proportional hazards regression analysis was done to determine predictors of survival from the time of gastrostomy insertion and from the time of disease onset (to take into account variables that have an effect on survival over the whole course of the disease). Our rationale for inclusion of covariates in the Cox regression analysis was based on well known factors that might affect survival in patients with amyotrophic lateral sclerosis as reported previously, and on our clinical judgment of other probable factors that might affect survival post gastrostomy. Continuity corrected χ^2^ tests were used to determine changes in nutritional status in the patients who underwent percutaneous endoscopic gastrostomy, radiologically inserted gastrostomy, or per-oral image-guided gastrostomy. Cox proportional hazards regression analysis was also done to examine the effect of nutritional status at 3 months post gastrostomy on subsequent survival. Cronbach's α coefficients were determined for the quality of life and strain measures used in the study to assess their internal consistency. A paired samples *t* test was used to determine differences in the self-perceived quality of life of the patients and the strain of caregiving activities of carers. We obtained complete mortality data for all patients who underwent gastrostomy. Initially, complete case analysis was done—ie, patients who had one or more missing values in the variables being analysed were omitted from the analysis pairwise. To compensate for missing data, post-hoc multiple imputation was done for the covariates of interest in our multiple regression analyses. Because ProGas was not a randomised controlled trial, we addressed the issue of treatment indication bias by undertaking a post-hoc propensity score analysis (appendix p 3).

Data were managed and analysed with SPSS Statistics for Windows version 21.0.

### Role of the funding source

This study was supported jointly by the Motor Neurone Disease Association of England, Wales, and Northern Ireland and the Sheffield Institute for Translational Neuroscience. Both funding bodies were consulted regarding the study design, and the decision to submit the report for publication fulfils their requirement for dissemination of the findings. However, the funding sources were not involved in data collection, data analysis, data interpretation, or writing of the report. All authors had full access to all of the data and CJM had final responsibility for the decision to submit the report for publication.

## Results

**Figure 1:**
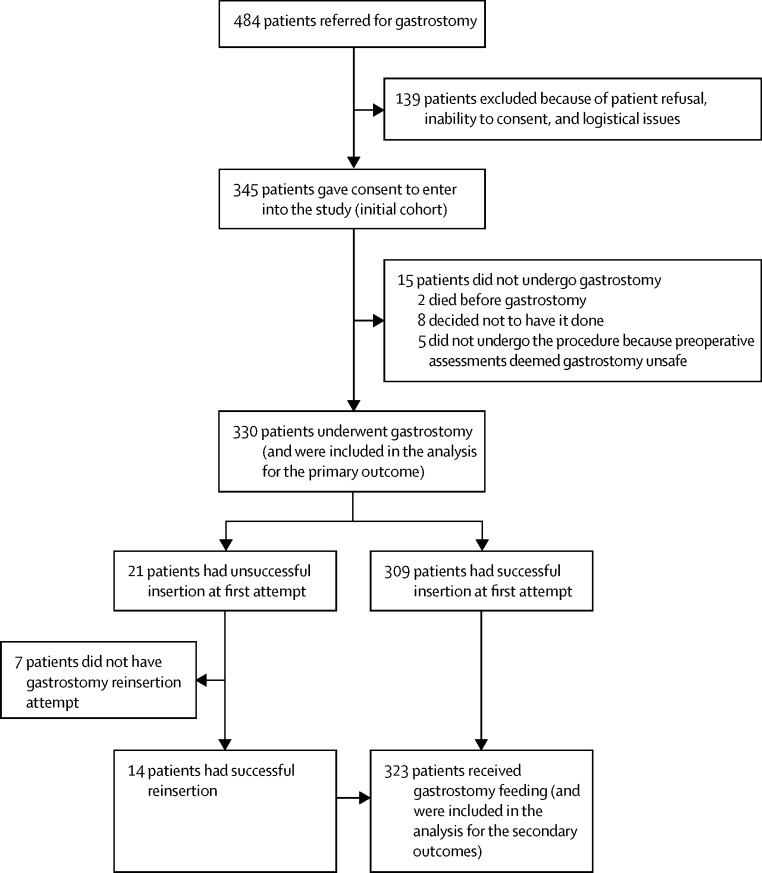
Study profile

Between Nov 2, 2010, and Jan 31, 2014, 330 patients underwent gastrostomy and were included in the analysis for the primary outcome (figure 1). Table 1 shows their baseline characteristics. 163 (49%) patients underwent percutaneous endoscopic gastrostomy, 121 (37%) underwent radiologically inserted gastrostomy, 43 (13%) underwent per-oral image-guided gastrostomy, and three (1%) underwent surgical gastrostomy. Table 2 summarises the differences across the three gastrostomy groups. Data for criteria used for gastrostomy method selection, indication, and predicted benefits on influence of patients on timing of gastrostomy, and on types and sizes of gastrostomy tubes are available on appendix p 1.

The study was funded for 38 months and stopped on Jan 31, 2013, at which point all patients had undergone data collection for the primary outcome. Nine patients did not undergo formal 3-month assessments and 93 patients did not undergo 12-month assessments.

12 (4%, 95% CI 2–6) of 330 patients died within the first 30 days after gastrostomy: five (3%, 1–7) of 163 after percutaneous endoscopic gastrostomy, four (3%, 1–8) of 121 after radiologically inserted gastrostomy, and three (7%, 2–19) of 43 after per-oral image-guided gastrostomy (p=0·46). We did not find evidence of a difference in 30-day mortality between the procedures after adjustment for case mix variables (age at onset, weight loss, functional decline rate, forced vital capacity, and site of onset) and treatment centre (appendix p 2).

**Figure 2:**
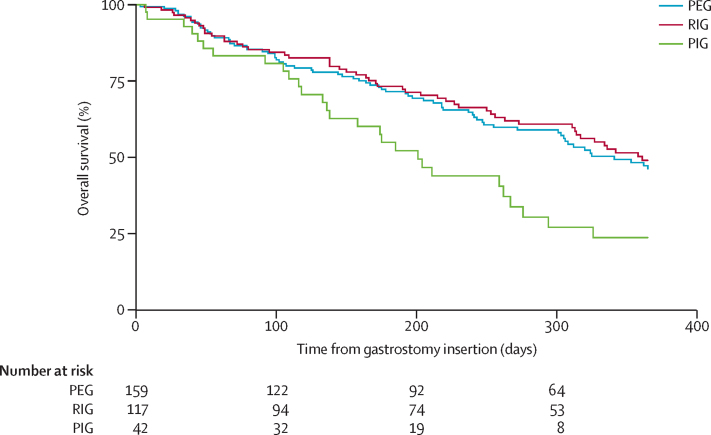
Survival functions for patients who underwent PEG, RIG, or PIG Subsequent Cox proportional hazards analysis suggested that the method of gastrostomy insertion was not significantly associated with survival. PEG=percutaneous endoscopic gastrostomy. RIG=radiologically inserted gastrostomy. PIG=per-oral image-guided gastrostomy.

Overall median survival after gastrostomy was 325 days (95% CI 289–361). Median survival time after percutaneous endoscopic gastrostomy was 341 days (25th IQR inderteminate–164), after radiologically inserted gastrostomy was 361 days (25th IQR inderteminate–171), and after per-oral image-guided gastrostomy was 201 days (326–116; figure 2). We noted some evidence of a difference in survival times (log-rank χ^2^ 1·4 on 2 df, p=0·03) between the three gastrostomy insertion methods before any adjustment for case mix variables (age at onset, weight loss, functional decline rate, forced vital capacity, and site of onset) and treatment centre. However, after adjustment for treatment centre and case mix variables, we did not note any evidence of a difference in survival times between the three gastrostomy insertion methods (appendix p 2).

Irrespective of method of gastrostomy, among the 12 patients who died within the first 30 days following the procedure, one patient (8%) had lost up to 10% of their bodyweight compared with that at diagnosis (2·6% loss), eight patients (67%) had lost more than 10% of their weight (mean 17·1% [SD 5·6]), one patient (8%) had gained weight (3·1% gain; χ^2^, n=252, p=0·031), and for two patients (17%) weight data were missing. Binary logistic regression analysis showed that the odds for 30-day mortality were 10·7 times higher (95% CI 1·3–87·0; p=0·027) for patients who had lost more than 10% of their weight from diagnosis compared with those who had lost 10% or less of weight.

Cox proportional hazards regression was done to ascertain the effect of gastrostomy method on survival from the time of gastrostomy insertion, with adjustment for covariates that might also affect survival. Variables that were inserted into the regression model were gastrostomy insertion method (percutaneous endoscopic gastrostomy, radiologically inserted gastrostomy, and per-oral image-guided gastrostomy subgroups), forced vital capacity at the time of gastrostomy insertion, percentage of weight difference at gastrostomy compared with diagnosis weight, and three additional well established predictors of survival in patients with amyotrophic lateral sclerosis:^19^ age at the onset of amyotrophic lateral sclerosis, site of amyotrophic lateral sclerosis symptom onset (bulbar and limb subgroups), and monthly rate of decline of the revised amyotrophic lateral sclerosis functional rating scale (ALSFRS-R). The results showed that the hazard of death after gastrostomy insertion was significantly affected by two main factors: the age at onset of amyotrophic lateral sclerosis (hazard ratio [HR] 1·032, 95% CI 1·007–1·059; p=0·013) and the percentage of weight difference at gastrostomy compared with weight at diagnosis (HR 0·956, 0·930–0·983; p=0·001). The hazard of death was not affected by the gastrostomy insertion method. Figure 2 shows the survival functions for the subgroups of patients who underwent percutaneous endoscopic gastrostomy, radiologically inserted gastrostomy, or per-oral image-guided gastrostomy.

A Cox proportional hazards regression model including the same variables showed that the hazard of death from the time of amyotrophic lateral sclerosis onset was significantly affected by the age at onset (HR 1·045 [95% CI 1·015–1·075]; p=0·003), the ALSFRS-R monthly decline rate (1·768 [1·541–2·028]; p=0·001), and the site of amyotrophic lateral sclerosis symptom onset (bulbar compared with limb subgroups, HR 2·082 [1·204–3·601]; p=0·009).

**Figure 3:**
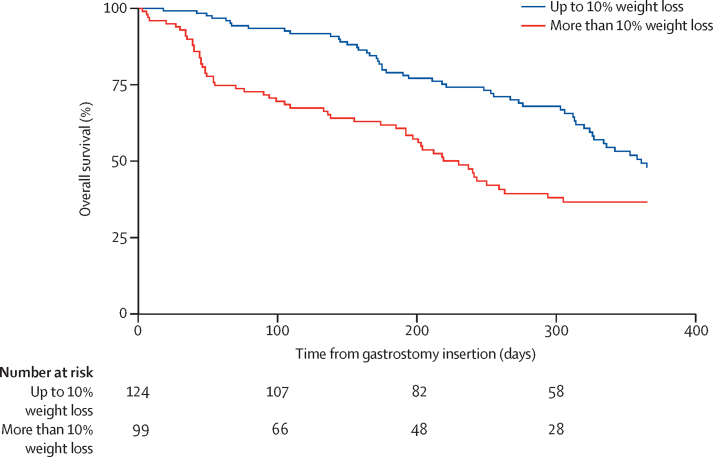
Survival functions according to weight loss

To further explore the effect of weight loss on survival after insertion, we did a Cox proportional hazards analysis with adjustment for covariates that might also affect survival. The regression model included as variables weight at the time of gastrostomy compared with weight at diagnosis (<10% weight loss and >10% weight loss subgroups), forced vital capacity at the time of gastrostomy insertion, age at the onset of amyotrophic lateral sclerosis, site of amyotrophic lateral sclerosis symptom onset (bulbar and limb subgroups), and ALSFRS-R monthly decline rate. Hazard of death after gastrostomy insertion was significantly affected by the age at onset (HR 1·035 [95% CI 1·008–1·063]; p=0·011) and the percentage of weight loss from diagnosis to gastrostomy (>10% weight loss subgroup compared with the <10% weight loss subgroup, 2·514 [1·490–4·243]; p=0·001). The median survival after gastrostomy for patients who had lost 10% or less of weight from diagnosis was 12 months (95% CI was indeterminate because survival was greater than 50% at the last timepoint in this subgroup) and for those who had lost more than 10% of their weight from diagnosis was 7·7 months (n=223, 95% CI 6·5–8·9; log-rank test p=0·001). Figure 3 shows the survival functions for the different subgroups of patients in terms of weight loss at gastrostomy compared with weight at the time of diagnosis.

Periprocedural complications did not differ significantly across the three gastrostomy insertion methods, apart from the higher perioperational distress experienced by percutaneous endoscopic gastrostomy patients (table 3). Table 4 summarises complications in the first 3 months after gastrostomy. Patients who received balloon-retention tubes (radiologically inserted gastrostomy) had a significantly higher rate of tube-related complications than did those who received bumper-retention tubes, including displacement (20 [31%] of 96 patients *vs* one [1%] of 154 patients; p=0·001), leakage (21 [22%] *vs* 16 [10%]; p=0·011), replacement (29 [30%] *vs* four [3%]; p=0·001), and repeated gastrostomy (14/96 [15%] *vs* one [1%]; 0·001; appendix p 1); percutaneous endoscopic gastrostomy and per-oral image-guided gastrostomy odds ratio data are given in appendix p 1).

Valid weight measurements at 3 months after gastrostomy were collected for 170 (53%) of 323 patients, owing to attrition and difficulty in obtaining weight measurements from wheelchair-bound patients. After gastrostomy insertion, 43 (25%) of 170 patients gained more than 1 kg compared with weight at gastrostomy, 43 (25%) had loss or gain of 1 kg or less compared with weight at gastrostomy, and 84 (49%) lost more than 1 kg compared to weight at gastrostomy. The method of gastrostomy insertion did not influence the bodyweight post procedure (χ^2^, n=170; p=0·082). In the 43 patients who gained weight, these gains were small (median weight gain compared with weight at gastrostomy 3·1 kg, IQR 1·8–6·5). Appendix p 6 shows the nutritional outcome for patients in terms of weight at 3 months compared with weight at diagnosis. Continuing weight loss at 3 months after gastrostomy was associated with poor survival (appendix p 2).

The differences between patient quality of life at baseline and 3 months after gastrostomy were not statistically significant (mean [SD] total MQOL score 6·3 [1·6] at baseline *vs* 6·4 [1·6] at 3 months; p=0·749; appendix p 2). However, the strain of caregiving activities had increased significantly for carers at 3 months after gastrostomy (mean [SD] total MCSI score 9·9 [6·4] at baseline *vs* 11·8 [6·5] at 3 months; p=0·001; appendix p 2).

The results of post-hoc multiple imputation and propensity score analyses of the survival endpoints suggested that our findings for 30-day mortality and predictors of survival were robust to both missing data and gastrostomy method preferences in the 24 participating sites (appendix pp 2, 3).

## Discussion

In our study, 30-day mortality was similar for percutaneous endoscopic gastrostomy, radiologically inserted gastrostomy, or per-oral image-guided gastrostomy, indicating that the three methods were as safe as each other in relation to procedure risk. The results suggested that weight loss at gastrostomy could affect procedure outcome, although caution with interpretation is necessary because 30-day mortality was low in this cohort. Our data indicate that overall mortality after gastrostomy insertion is independent of the gastrostomy method and is driven by the patient age at the onset of amyotrophic lateral sclerosis and the percentage of weight loss from diagnosis to the timepoint of gastrostomy.

In terms of periprocedural complications, the three different methods of gastrostomy were similar apart from the increased rate of distress, related to procedure tolerance, experienced by patients who underwent percutaneous endoscopic gastrostomy. This finding can be explained by the nature of the percutaneous endoscopic gastrostomy procedure, during which the patient's throat is intubated with an endoscope and the gastrostomy tube is pulled through the mouth towards its placement site.^20^ Patients who underwent radiologically inserted gastrostomy had a significantly increased rate of gastrostomy tube-related complications. Perhaps this is not surprising, because radiologically inserted gastrostomy tubes are usually relatively narrow in diameter (10–14 Fr), have a balloon-retention system (balloons could burst or deflate, causing gastrostomy tubes to migrate or fall out), and are not as securely fixed as those inserted by percutaneous endoscopic gastrostomy or per-oral image-guided gastrostomy.

In terms of the nutritional outcome for the patient, gastrostomy feeding prevented further weight loss in only about half of the patients. In the 43 (25%) patients who gained weight, these gains were small and of doubtful clinical benefit. Continuing weight loss at 3 months after gastrostomy was associated with poor survival. The nutritional data suggested that the greater the percentage of weight loss at the time of gastrostomy from diagnosis, the less likely it was for patients to recover this loss after gastrostomy. This finding was more evident for patients who at the time of gastrostomy had had more than 10% loss of their diagnosis weight; this subgroup of patients had also a significantly shorter survival compared with those who had lost up to 10% of their diagnosis weight. These results suggest that patients might benefit from early gastrostomy, before substantial weight loss that might not be reversible.

The reasons for the fairly poor nutritional outcome that we noted need further investigation. Perhaps weight loss due to continued denervation-induced skeletal muscle atrophy is masking nutritional benefits,^21,22^ which could be related to the change in metabolic state. Patients with amyotrophic lateral sclerosis can present hypermetabolism, and the caloric requirements of patients after gastrostomy might have been underestimated such that their energy intake was lower than energy expenditure.^23^ A small phase 2 study showed a potential benefit in terms of survival and nutritional gains for patients fed high calorific diets through a percutaneous endoscopic gastrostomy tube.^24^ Therefore, further study and subsequent evidence-based guidance on nutritional management post gastrostomy tube insertion is needed. A further potential metabolic explanation for our findings is related to the concept of refractory cachexia. The body of a patient with cachexia (defined as weight loss of more than 5%) is recognised to undergo irreparable metabolic changes, making artificial nutritional support ineffective.^25^ This idea is well recognised in oncology but has not been explored in patients with amyotrophic lateral sclerosis.

The effect of gastrostomy on the quality of life of patients in our study seemed to be neutral. Conversely, the strain of caregiving activities increased significantly after gastrostomy, although this was independent of insertion method. However, consequences of amyotrophic lateral sclerosis including increasing motor disability and dependency might contribute to caregiver strain. These results highlight the importance of provision of information and support from health-care professionals to carers, as well as to patients, before and after gastrostomy.

This study has limitations. This study was not a randomised controlled trial and the assignment of patients to a specific gastrostomy method was not done at random, but based on practical and clinical considerations. Therefore we can make associations but we are limited in the ability to draw conclusions with regard to the direct effects of gastrostomy on survival and nutritional outcome compared with not having had a gastrostomy. Another limitation is that, of 484 patients who had been referred for a gastrostomy in the 24 participating centres, we recruited 345 patients (participation rate 71%). Patient refusal and several logistical issues hindered full recruitment. Unfortunately, we could not obtain meaningful information for the potential participants who were not recruited to our study because we did not have the consent of these patients to do so, and we could not compare their characteristics with those of patients in this study. Practical difficulties in obtaining weight measurements at 3 months after gastrostomy introduced another limitation. The prospective element of this study allowed us to follow up a large number of patients after gastrostomy insertion and to consistently collect data related to the predetermined primary and secondary outcomes. A major strength of this study is that our sample is representative of the wider amyotrophic lateral sclerosis population: the baseline characteristics of the patients who took part are very similar to those of other reported cohorts of patients with amyotrophic lateral sclerosis.^19,26^

We noted significantly worse respiratory impairment in the per-oral image-guided gastrostomy group. Despite this, 30-day mortality was similar to the other groups. This observation would suggest that percutaneous endoscopic gastrostomy might be the optimum method of gastrostomy when respiratory function is largely unimpaired and per-oral image-guided gastrostomy when respiratory function is significantly compromised. Both percutaneous endoscopic gastrostomy and per-oral image-guided gastrostomy seemed to offer easier post-insertion tube management than radiologically inserted gastrostomy; ease of management is crucial, especially in very frail patients who undergo gastrostomy late, when they are more likely to feel the burden of other consequences of amyotrophic lateral sclerosis, such as respiratory problems and the loss of mobility and speech.

Our study showed that delay might lead to diminishing gains, especially for patients who at the time of gastrostomy have experienced excessive weight loss from their diagnosis weight. From a safety and efficacy perspective, the current guidelines of 10% weight loss might not be ideal and perhaps a better threshold would be to recommend gastrostomy insertion at a threshold similar to the one for cachexia—ie, at roughly 5% weight loss. Another recently suggested approach is to consider gastrostomy based on the ability of an individual to meet their total daily energy requirements.^27^ Delay of gastrostomy until after weight loss of more than 10% might convey minimal clinically meaningful benefit. However, some patients will not wish to undergo early gastrostomy. For such patients, gastrostomy will still have a role alleviating the difficulties caused by advanced dysphagia—eg, to allow administration of drugs and hydration—but in view of the possible diminishing nutritional benefits of delayed gastrostomy, other options of palliative support should also be considered.

Correspondence to: Dr Christopher J McDermott, Sheffield Institute for Translational Neuroscience, The University of Sheffield, 385A Glossop Road, Sheffield, S10 2HQ, UK **c.j.mcdermott@sheffield.ac.uk**

## Figures and Tables

**Table 1:** Baseline demographic and clinical characteristics

		**Baseline value in all patients (n=330)**
Age (years)		64·4 (11·7), n=315
Sex		
	Women	150/330 (45%)
	Men	180/330 (55%)
Forced vital capacity (%)		62% (22·6), n=258
% Weight loss from diagnosis to baseline (kg)		8·6 (9·8), n=252
ALSFRS-R score		28 (8·5), n=307
Body-mass index (kg/m^2^)		23·3 (4·4), n=274
Monthly ALSFRS-R decline		2·2% (1·7%), n=290
Disease duration from diagnosis (months)		16·7 (5·8–14·9), n=309
Non-invasive ventilation routine users		81/323 (25%)
Site of disease onset		
	Limb	152/324 (47%)
	Bulbar	165/324 (51%)
	Both limb and bulbar	6/324 (2%)
	Respiratory	1/324 (0·3%)

Data are mean (SD), median (IQR), or n/N (%). ALSFRS-R=amyotrophic lateral sclerosis functional rating scale revised.

**Table 2:** Baseline differences of patients who underwent PEG, RIG, or PIG

		**PEG**	**RIG**	**PIG**	**Statistic (n)**	**p value**
Age (years)		64·2 (11·7), n=157	63·6 (9·8), n=114	67·2 (12·6), n=41	F (312)	0·200
Sex					χ^2^ (327)	0·683
	Women	73/163 (45%)	59/121 (49%)	18/43 (42%)		
	Men	90/163 (55%)	62/121 (51%)	25/43 (58%)		
FVC (%)		65·4 (22·2), n=136	59 (23·1), n=87	52 (19·7), n=33	F (256)	0·004
% Weight loss from diagnosis to baseline (kg)		7·1 (8·5), n=117	8·7 (9·9), n=98	13 (12·3), n=35	F (250)	0·008
ALSFRS-R score		29·1 (8·2), n=152	27·7 (8·8), n=114	24·7 (7·9), n=39	F (305)	0·014
Body-mass index (kg/m^2^)		23·7 (4), n=135	23·4 (5·1), n=102	21·8 (3), n=34	F (271)	0·091
Monthly ALSFRS-R decline		2·1% (1·5), n=144	2·4% (2·1), n=105	2·1% (1·2), n=39	F (288)	0·302
NIV routine users		29/162 (18%)	23/118 (19%)	28/42 (67%)	χ^2^ (322)	0·001
Site of disease onset					χ^2^ (321)	0·369
	Limb	74/161 (46%)	54/117 (46%)	23/43 (53%)		
	Bulbar	86/161 (53%)	59/117 (50%)	18/43 (42%)		
	Both limb and bulbar	1/161 (1%)	3/117 (3%)	2/43 (5%)		
	Respiratory	0/161	1/117 (1%)	0/43		

Data are mean (SD) or n/N (%). PEG=percutaneous endoscopic gastrostomy. RIG=radiologically inserted gastrostomy. PIG=per-oral image-guided gastrostomy. F=one-way ANOVA F test. FVC=forced vital capacity. ALSFRS-R=amyotrophic lateral sclerosis functional rating scale revised. NIV=non-invasive ventilation.

**Table 3:** Periprocedural complications

	**PEG**	**RIG**	**PIG**	**Total**	**p value**
Overall complication rate	41/169 (24%)	20/121 (17%)	8/42 (19%)	69/332 (21%)	0·266
Difficult procedure	26/168 (16%)	13/119 (11%)	7/42 (17%)	46/329 (14%)	0·475
Failed attempt	11/171 (6%)	7/125 (6%)	3/45 (7%)	21/341 (6%)	0·947
O_2_desaturation	6/166 (4%)	2/117 (2%)	3/42 (7%)	11/325 (3%)	0·241
Patient distress	26/166 (16%)	4/117 (3%)	2/42 (5%)	32/325 (10%)	0·002
Respiratory arrest	0/166	0/117	0/42	0/325	NA
Laryngeal spasm	2/166 (1%)	1/117 (1%)	0/42	3/325 (1%)	0·763
Haemorrhage	0/166	3/117 (3%)	0/42	3/325 (1%)	0·068

Numbers are patients who experienced each event (n)/total patients in each group (N). The periprocedural period is the time during the gastrostomy procedure. PEG=percutaneous endoscopic gastrostomy. RIG=radiologically inserted gastrostomy. PIG=per-oral image-guided gastrostomy. NA=not applicable.

**Table 4:** Postprocedural complications

	**PEG**	**RIG**	**PIG**	**Total**	**p value**
Infection	20/129 (16%)	21/96 (22%)	3/25 (12%)	44/250 (18%)	0·745
Granulation tissue	15/129 (12%)	19/96 (20%)	3/25 (12%)	37/250 (15%)	0·214
Pain	25/129 (19%)	34/96 (35%)	10/25 (40%)	69/250 (28%)	0·010
Anxiety	10/129 (8%)	24/96 (25%)	1/25 (4%)	35/250 (14%)	0·001
Nausea	12/129 (9%)	10/96 (10%)	2/25 (8%)	24/250 (10%)	0·923
Diarrhoea	6/129 (5%)	10/96 (10%)	3/25 (12%)	19/250 (8%)	0·185
Pneumonia	4/129 (3%)	4/96 (4%)	4/25 (16%)	12/250 (5%)	0·021
Constipation	16/129 (13%)	22/96 (24%)	9/25 (36%)	47/250 (19%)	0·010
Fatigue	15/129 (12%)	23/96 (24%)	4/25 (16%)	42/250 (17%)	0·050

Numbers are patients who experienced each event (n)/total patients in each group (N). The postprocedural period is the first 3 months after the completion of the gastrostomy procedure. PEG=percutaneous endoscopic gastrostomy. RIG=radiologically inserted gastrostomy. PIG=per-oral image-guided gastrostomy.
