# Supplementary material for: Gastrostomy in patients with amyotrophic lateral sclerosis (ProGas): a prospective cohort study
Source: Lancet Neurol. 2015 Jul;14(7):702–9. doi: 10.1016/S1474-4422(15)00104-0 (PMC4578147; doi:10.1016/S1474-4422(15)00104-0)
Supplement: Supplementary appendix [file mmc1.pdf]

THE LANCET Neurology

Supplementary webappendix

This webappendix formed part of the original submission and has been peer reviewed. We post it as supplied by the authors.

Supplement to: ProGas Study Group. Gastrostomy in patients with amyotrophic lateral sclerosis (ProGas): a prospective cohort study. *Lancet Neurol* 2015; published online May 29. http://dx.doi.org/10.1016/S1474-4422(15)00104-0.

Supplementary data

Gastrostomy method availability
PEG was performed in 17, RIG in 18 and PIG in 3 sites. Of all 24 sites, 12 had access to both endoscopic (PEG) and radiological (RIG and/or PIG) gastrostomy services. 5 sites had access only to an endoscopic gastrostomy service, whereas 7 sites had access only to a radiological gastrostomy service. One site had access to all three different methods of gastrostomy and in another site PEG was performed with non-invasive ventilation (NIV) support if necessary.

Criteria for gastrostomy method selection
The clinicians were asked to state their reasons for referring patients for a specific gastrostomy method. In sites where both endoscopic and radiological methods of gastrostomy insertion were available, there was a clear clinician preference to refer for a PEG procedure patients with good respiratory function and who were predicted to be able to tolerate endoscopy (i.e., able to lie flat, receive sedation). PEG was considered by some clinicians as the easier pathway, being quicker to perform and resulting in an easier to use tube, as well as simpler patient management after insertion. RIG and PIG methods were preferred for patients who were more frail, with compromised respiratory function and when the risk of endoscopy was considered to be high.

Indication for gastrostomy
The most commonly reported indications for gastrostomy were marked weight loss in 195/339 (57·5%) patients; unsafe swallow in 201/339 (59·3%) patients; and prolonged difficult meals in 214/339 (63·1%) patients. Recurrent aspiration in 32/339 (9·4%) patients; anticipation of a future need in 20/344 (5·8%) patients; worsening swallow in 9/334 (2·6%) patients; worsening breathing in 8/344 (2·3%) patients; and anorexia in 7/344 (2%) patients were less frequent indications. Other infrequently reported indications were air swallowing, frequent coughing on attempts to swallow, NIV mask causing eating difficulties, severe secretion difficulties, problems opening the mouth to eat and difficulty in feeding due to limb weakness.

Predicted benefits of gastrostomy
The clinicians perceived that gastrostomy would stabilise the nutrition and hydration for 294/337 (87·2%) patients; ease the feeding difficulties for 265/337 (78·6%) patients; reduce the risk of choking and improve the quality of life for 261/337 (77·4%) patients; and provide an alternative route for medication for 237/337 (70·3%). A potential benefit of reduced aspiration and chest infection risk was perceived for nearly two thirds of patients 219/337 (65%). Prolonging survival was perceived for less than half of patients 161/337 (47·8%) and reduction of carer burden for 150/337 (44·5%) of the informal carers of patients.

Timing of gastrostomy
With regard to timing of gastrostomy, clinicians reported that 47/328 (14·3%) patients deliberately delayed undergoing gastrostomy insertion, after it had been recommended. The stated reasons were patient reluctance to give up oral feeding; willingness to carry on coping with the difficulties of dysphagia for longer; time needed to adjust to the idea of gastrostomy; time needed to contemplate involved risks and potential benefits of gastrostomy; worrying about the procedure; personal and family circumstances; and negative perception of gastrostomy, such as the idea that gastrostomy signifies "the beginning of the end".

Types and sizes of gastrostomy tubes
The type and size of the gastrostomy tubes used across the three different subgroups of patients varied. PEG and PIG patients received similar large bore bumper-retention gastrostomy tubes placed with the 'pull-through' technique. 2/157 (1·3%) PEG patients received size 12 Fr; 1/157 (0·6%) 14 Fr; 79/157 (50·3%) size 15 Fr; 51/157 (32·5%) size 16 Fr and 21/157 (15·3%) size 20 Fr gastrostomy tubes. 8/40 (20%) PIG patients received size 15 Fr; and 32/40 (80%) size 16 Fr gastrostomy tubes. RIG patients received smaller bore balloon-retention gastrostomy tubes. 6/110 (5·4%) RIG patients received size 10 Fr; 72/110 (65·5%) size 12 Fr; 26/110 (23·6%) size 14 Fr; and 6/110 (5·5%) size 16 Fr gastrostomy tubes.

Tube-related complications
49/96 (51·0%) patients who received balloon-retention tubes (RIG) experienced a significantly higher rate of complications related to tube management in the first three months following gastrostomy (Odds Ratio balloon-to-bumper=2·397, 95% CI 1·180-4·870, p=0·015); tube displacement (Odds Ratio balloon-to-bumper=40·263, 95% CI 5·303-305·683, p=0·001); tube replacement (Odds Ratio balloon-to-bumper=15·049, 95% CI 5·081-44·575, p=0·001); and repeated gastrostomy as a consequence of tube dislodgment (Odds Ratio balloon-to-bumper = 24·198, 95% CI 3·124-187·424, p=0·001), compared to 21/154 (13.6%) patients who received bumper-retention tubes. Pain (Odds Ratio balloon-to-bumper=1·865, 95% CI 1·062-3·274, p=0·027), increased anxiety

(Odds Ratio balloon-to-bumper=4·333, 95% CI 2·011-9·338, p=0·001) and fatigue (Odds Ratio balloon-to-bumper=2·239, 95% CI 1·144-4·379, p=0·018) was also significantly higher for patients who received balloon-retention tubes.

Influence of nutritional status at three months on subsequent survival
We were interested in the influence of nutritional status at three months on the subsequent survival. A Cox proportional-hazards analysis was performed to ascertain the predictors which influenced survival. The variables that were included in the regression model were: weight groups at three months following gastrostomy, compared to diagnosis weight (no weight loss, <10% weight loss and >10% weight loss subgroups); FVC at the time of gastrostomy; age at the onset of MND; site of MND symptom onset (bulbar and limb subgroups) and ALSFRS-R monthly decline rate. The results demonstrated that the hazard of death was significantly influenced by the percentage of weight loss from diagnosis at three months following gastrostomy insertion (Hazard Ratio >10% weight loss group to No weight loss group=3·802, 95% CI 1·127-12·820, p=0.031; and Hazard Ratio >10% weight loss group to <10% weight loss group= 2·717, 95% CI 1·331-5·555, p=0.006). The age at the onset of MND also significantly influenced survival (Hazard Ratio=1·038, 95% CI 1·002-1·077, p=0.041).

Impact on quality of life
Patient response to the MQOL at baseline was 283/321 (88·2%) and at 3 months following gastrostomy insertion was 162/321 (50·5%). The internal consistency of the MQOL questionnaires that were included in the analysis was satisfactory (baseline and 3-month MQOL Cronbach's α=0·8). The mean (SD) total MQOL score at baseline was 6·3 ± 1·6 and at 3 months following gastrostomy was 6·4 ±1·6 (p=0·749). Compared to pre-gastrostomy, patient quality of life appeared unchanged in 24/114 (21·1%) patients, improved in 42/114 (36·8%), and deteriorated in 48/114 (42·1%). A continuity chi-square test was performed to determine the difference between patients (n=114) in the three quality of life subgroups and gastrostomy method. The differences were not significant (p=0.122) suggesting that quality of life following gastrostomy was not influenced by gastrostomy insertion method.

In total, 233 carers were initially recruited into ProGas. Carer response to the MCSI at baseline was 207/233 (88·8) and at 3 months following patient's gastrostomy insertion was 114/233 (48·9%). The internal consistency of the MCSI questionnaires that were included in the analysis was satisfactory (baseline and 3-month MCSI Cronbach's α=0·9). The mean (SD) total MCSI score at baseline was 9·9 ±6·4 and at 3 months following gastrostomy was 11·8 ±6·5 (p=0·001). Compared to pre-gastrostomy, the strain of caregiving activities remained the same in 17/97 (17·5%); increased in 56/97 (57·7%); and decreased in 24/97 (24·7%) of carers. A continuity chi-square test was performed to determine the difference between carers (n=97) in the three caregiving strain subgroups and gastrostomy method. The differences were not significant (p=0.558) suggesting that caregiving strain was not influenced by gastrostomy insertion method.

Treating centre effect adjustment for 30-day mortality following gastrostomy
A binary logistic regression analysis was performed to determine the differences in 30-day mortality rate between PEG, RIG and PIG patients adjusting for treating centre and other covariates that may influence survival following gastrostomy, such as age at onset of MND; percentage of weight loss from diagnosis; ALSFRS-R monthly decline rate; FVC; and site of MND symptom onset. The results demonstrated that the differences were not significant (Table 1).

Treating centre effect adjustment for median survival time following gastrostomy
A Cox proportional-hazards analysis was performed to determine the differences in median survival time between PEG, RIG and PIG patients adjusting for treating centre and other covariates that may influence survival following gastrostomy, such as age at onset of MND; percentage of weight loss from diagnosis; ALSFRS-R monthly decline rate; FVC; and site of MND symptom onset. The results demonstrated that the differences were not significant (Table 2).

Imputation analysis
For the primary outcome variable, i.e., 30-day mortality following gastrostomy, we had no missing values. However, we did have missing values for the covariates (i.e., percentage of weight loss from diagnosis, FVC ALSFRS-R monthly decline rate, age at onset of MND, site of MND symptom onset, NIV routine use) that we included in our multiple regression models. To compensate for the missing data we performed multiple imputation (MI) analysis, which involves generating multiple completed datasets for analysis by replacing missing values with plausible ones based on simulation models.[1]
First, we examined the extent of missing values in the six covariates and found that 238/1980 (12%) of all values were missing. Specifically, for the covariate percentage of weight loss from diagnosis 78/330 (23·6%)

values were missing; for FVC 72/330 (21·8%) values were missing; for ALSFRS-R monthly decline rate 40/330 (12·1%) values were missing; for site of MND symptom onset 13/330 (3·9%) values were missing; and for NIV routine use 7/330 (2·1%) values were missing. There was no evidence of monotonicity in the patterns of missing values in the six covariates, indicating that the missing values were probably missing in a random pattern.

MI is an iterated process; hence, we set the random seed in our statistical software (SPSS v.22), i.e., set the parameters for SPSS to randomly generate numbers to create the iterations for the missing values. We used the Mersenne Twister, a random number generator programme, and set the default fixed value of 2000000 as a starting point. We then performed the multiple imputation using the Markov Chain Monte Carlo algorithm. Based on the percentage of missing values in our covariates five imputations were sufficient to give an efficiency of 97%.[2] 100 iterations were used for each simulation to increase the likelihood of attaining convergence, i.e., ensure that the imputations were independent.[3]

Having generated the imputed data we repeated the multiple regression models with the same covariates reported in the main text of the paper. The results of the MI analysis were consistent with those of the complete case analysis. In relation to our primary outcome, there was no evidence of a difference in 30-day mortality between the three gastrostomy insertion methods after adjustment for case mix variables (Table 3). In relation to survival following gastrostomy insertion, there was no evidence of a difference in survival times between the three gastrostomy insertion methods after adjustment for case mix variables (Tables 4, 5 and 6).

Propensity score analysis

We have estimated three propensity scores for each three treatments (the predictive probability of having each of the three methods of gastrostomy insertion given the covariates) using a multinomial logistic regression model with age at onset of MND; % of weight loss from diagnosis; ALSFRS- R monthly decline rate; FVC; and site of MND symptom onset as predictor covariates of treatment. Since we have three treatments/methods of gastrostomy insertion which results in three probabilities or propensity scores we have used a simple propensity score covariate adjustment (PS-CA) method rather than more complex inverse probability weighting estimation or matching (using neighbour matching). Since the three propensity scores will sum to unity; we have used two of the propensity scores as covariates (PS-CA) along with treatment (method of insertion) in binary logistic regression model to compare the effect of treatment on 30-day mortality. Since we have missing covariates (age at onset of MND, % of weight loss from diagnosis, ALSFRS-R monthly decline rate, and FVC) for the estimation of the propensity scores we imputed these missing covariates by simple mean imputation stratified by treatment group and repeated the propensity score analysis using the imputed data. The results of this analysis are presented in Table 3.

Tables

**Variable**	***p* value**
Age at onset of MND	0·535
% of weight loss from diagnosis	0·911
FVC	0·328
ALSFRS-R monthly decline rate	0·543
Site of MND symptom onset	0·993
Gastrostomy insertion method	1·000
Treating centre	1·000

Table 1: Results of *p* values for all covariates included in the binary logistic regression to determine the differences in 30-day mortality rate between gastrostomy method, adjusting for treating centre and other covariates that may influence post-gastrostomy survival

**Variable**	***p* value**
Age at onset of MND	0·003
% of weight loss from diagnosis	0·004
FVC	0·051
ALSFRS-R monthly decline rate	0·625
Site of MND symptom onset	0·641
Gastrostomy insertion method	0·921
Treating centre	0·088

Table 2: Results of *p* values for all covariates included in the cox proportional-hazards regression model to determine the differences in post-gastrostomy median survival time between gastrostomy method, adjusting for treating centre and other covariates that may influence post-gastrostomy survival

Variables in the model	N	Category group	Odds Ratio	95%CI	*p* value
**(Model 1 – complete case data; unadjusted for covariates)**					
Method of gastrostomy insertion only	327	PEG			
		RIG vs PEG	1·08	0·28 – 4·11	0·91
		PIG vs PEG	2·37	0·54 – 10·34	0·251
**(Model 2 – complete case data; adjusted for covariates)**					
Method of gastrostomy insertion; age at onset of MND; % of weight loss from diagnosis; ALSFRS-R monthly decline rate; FVC; site of MND symptom onset	172	PEG			
		RIG vs PEG	2·71	0·21 – 34·22	0·441
		PIG vs PEG	5·00	0·36 – 69·10	0·23
**(Model 3 – Imputation of missing covariates; adjusted for covariates)**					
Method of gastrostomy insertion; age at onset of MND; % of weight loss from diagnosis; ALSFRS-R monthly decline rate; FVC; site of MND symptom onset	327	PEG			
		RIG vs PEG	1·146	0·279 – 4·713	0·85
		PIG vs PEG	1·596	0·314 – 8·106	0·572
**(Model 4 – Propensity scores using observed data)**					
Method of gastrostomy insertion and propensity scores*	172	PEG			
		RIG vs PEG	2·59	0·22 – 30·56	0·45
		PIG vs PEG	4.93	0·37 – 65·92	0·228
**(Model 5 – Propensity scores using imputation for missing data in the covariates)**					
Method of gastrostomy insertion and propensity scores with imputed missing covariates**	327	PEG			
		RIG vs PEG	0·98	0·25 – 3·90	0·981
		PEG vs PEG	1·39	0·28 – 6·94	0·686

Table 3: Results of sensitivity analysis to determine the effect of gastrostomy method on the 30-day mortality following gastrostomy insertion.

*The three propensity scores for each three treatments (the predictive probability of having each of the three methods of gastrostomy insertion given the covariates) were estimated using a multinomial logistic regression model with age at onset of MND; % of weight loss from diagnosis; ALSFRS-R monthly decline rate; FVC; and site of MND symptom onset as predictor covariates of treatment. We used two of the propensity scores as covariates along with treatment (method of gastrostomy insertion) in a binary logistic regression model to compare the effect of treatment on 30-day mortality.
**Missing covariates (age at onset of MND, % of weight loss from diagnosis, ALSFRS-R monthly decline rate and FVC) were estimated by simple mean imputation stratified by treatment group.

Variable (Reference category group)	Category group	Risk Ratio	95%CI	*p* value
Age at onset of MND		1·023	1·005-1·040	0·012
% of weight loss from diagnosis		0·968	0·951-0·986	0·001
ALSFRS-R monthly decline rate		1·063	0·978-1·157	0·152
FVC		0·993	0·986-1·001	0·069
Gastrostomy method (PEG patient group)	RIG patient group	0·820	0·564-1·192	0·298
	PIG patient group	1·364	0·853-2·181	0·195
Site of MND symptom onset (limb onset)	Bulbar onset	0·944	0·662-1·348	0·752

Table 4: A Cox proportional-hazards regression model, with imputed data, showing the pooled effect of gastrostomy method and other predictors of survival following gastrostomy insertion on the risk of death after gastrostomy (n=320).

Variable (Reference category group)	Category group	Risk Ratio	95%CI	*p* value
Age at onset of MND		1·033	1·015-1·052	0·001
% of weight loss from diagnosis		0·987	0·968-1·006	0·173
ALSFRS-R monthly decline rate		1·570	1·414-1·744	0·001
FVC		0·995	0·987-1·002	0·174
Gastrostomy method (PEG patient group)	RIG patient group	0·988	0·634-1·538	0·995
	PIG patient group	1·305	0·801-2·126	0·284
Site of MND symptom onset (limb onset)	Bulbar onset	2·235	1·476-3·385	0·001

Table 5: A Cox proportional-hazards regression model, with imputed data, showing the pooled effect of gastrostomy method and other predictors of survival from the time of disease onset on the risk of death after gastrostomy (n=320).

Variable (Reference category group)	Category group	Risk Ratio	95%CI	*p* value
Age at onset of MND		1·024	1·003-1·045	0·022
ALSFRS-R monthly decline rate		1·023	0·922-1·135	0·670
FVC		0·998	0·990-1·006	0·631
% of weight loss from diagnosis (>10% weight loss group)	<10% weight loss group	2·106	1·409-3·147	0·001
Site of MND symptom onset (limb onset)	Bulbar onset	0·862	0·558-1·333	0·505

Table 6: A Cox proportional-hazards regression model, with imputed data, showing the pooled effect of weight loss from diagnosis and other predictors of survival from on the risk of death after gastrostomy (n=320)

Figures

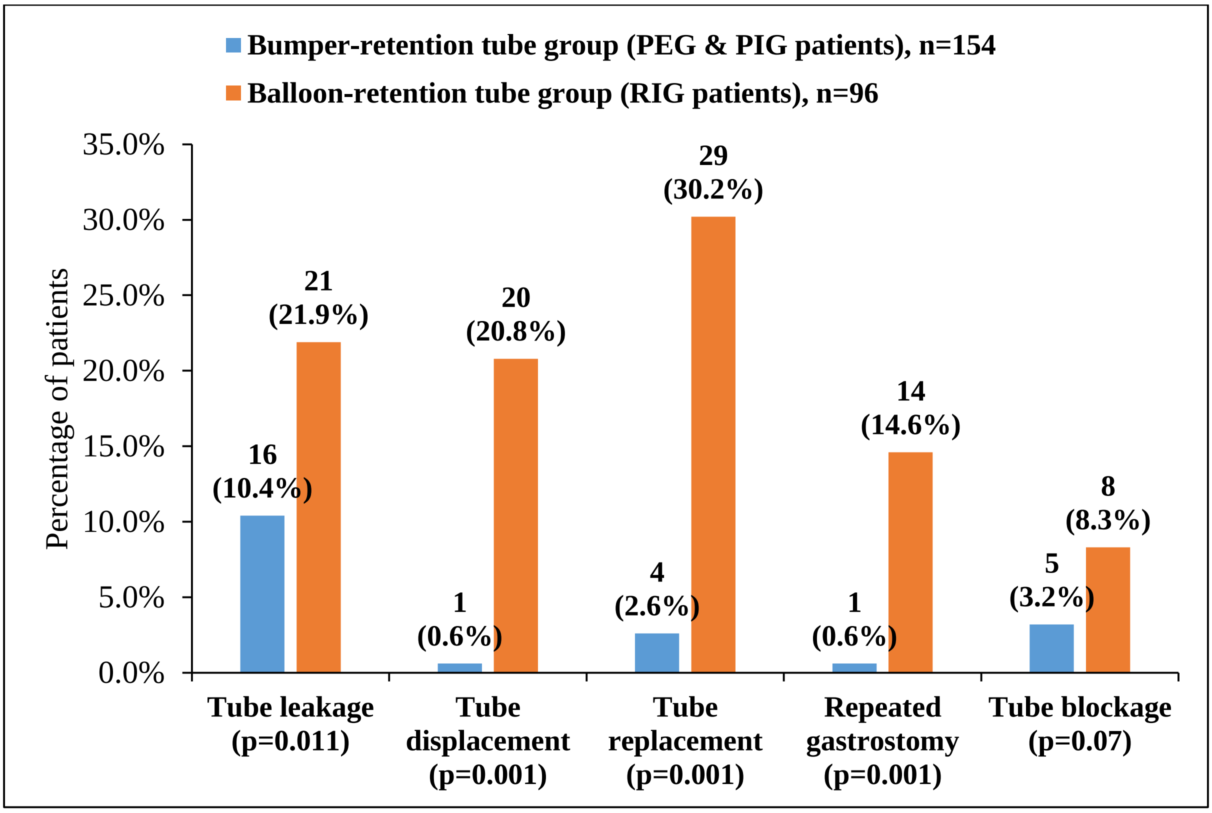

Figure 1: Tube-related complications in the first three months following gastrostomy insertion in terms of tube retention type.

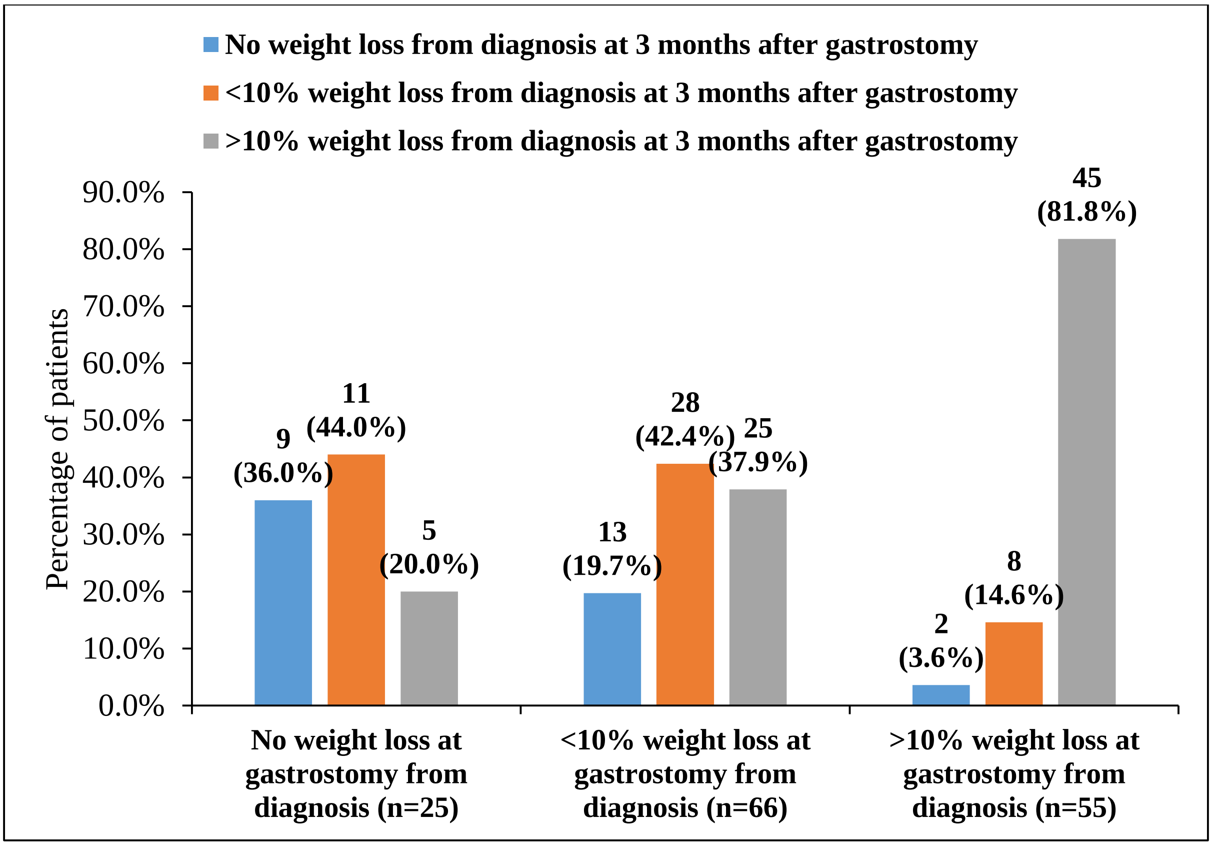

Figure 2: Nutritional outcome for patients in terms of weight at three months post-gastrostomy compared to weight at diagnosis

References

1. Rubin DB. Multiple imputation for nonresponse in surveys. New York; 1987
2. Graham JW, Olchowski AE, Gilreath TD. How many imputations are really needed? Some practical clarifications of multiple imputation theory. *Prev Sci* 2007; 8: 206-13.
3. Schafer JL. Multiple imputation: a primer. *Stat Methods Med Res* 1999; 8: 3-15.

Collaborators

Basildon University Hospital, Basildon: A Malaspina (principal investigator), E Mills; *Royal Victoria Hospital, Belfast*: C Donaghy (principal investigator), B McLaughlin; *Queen Elizabeth Hospital Birmingham, Birmingham*: K Morrison (principal investigator), H Jew; *Southern General Hospital, Glasgow*: G Gorrie (principal investigator); *King's College Hospital, London*: A Al-Chalabi (principal investigator), S Chapman, A Dougherty; *Leeds General Infirmary, Leeds*: M Johnson (principal investigator), L Tuttle; *The Walton Centre, Liverpool*: C Young (principal investigator), L Patel; *Salford Royal Hospital, Salford*: H Hamdalla (principal investigator), J Ealing, J Maskell, R Morris, L Howell; *Newcastle General Hospital, Newcastle upon Tyne*: T Williams (principal investigator), S Dodds; *Norfolk and Norwich University Hospital, Norwich*: D Dick (principal investigator), A Hursey; *Queen's Medical Centre, Nottingham*: C Gent (principal investigator); *John Radcliffe Hospital, Oxford*: K Talbot (principal investigator), M R Turner (principal investigator), M Lord, R Marsden; *Derriford Hospital, Plymouth*: C O Hanemann (principal investigator), S Angus, T Thomas; *Royal Preston Hospital, Preston*: T Majeed (principal investigator), A Kennedy, T Shaw, P Callagher, W Bennett, R Addison-Jones, A Parkes; *Royal Free Hospital, London*: R Orrell (principal investigator), J Bennell; *Royal London Hospital, London*: A Radunovic (principal investigator), A Malaspina (principal investigator), E Mills; *Sheffield Institute for Translational Neuroscience (SITraN), University of Sheffield, Sheffield*: C J McDermott (chief investigator and study manager), P J Shaw (co-chief investigator), T Stavroulakis (co-investigator), T Walsh; *School of Health and Related Research (ScHARR), University of Sheffield, Sheffield*: W O Baird (co-investigator), S J Walters (statistician); *Royal Hallamshire Hospital, Sheffield Teaching Hospitals NHS Foundation Trust, Sheffield*: M McAlindon (co-investigator), F Lee (co-investigator), S Webber (co-investigator), A Glossop (co-investigator); *Southampton General Hospital, Southampton*: A Pinto (principal investigator), V Agarwal, C Erridge; *West Suffolk Hospital, Bury St Edmunds*: F Crawley (principal investigator), L Canovas; *Centre for Neuroregeneration, Edinburgh*: S Chandran (principal investigator), S

Colville, L Stephenson; *Wythenshawe Hospital*, *Manchester*: M Roberts (principal investigator), L Harpin, D Martin, J Maskell, E Oughton, A Howman, A Davies; *Leicester General Hospital*, *Leicester*: P Eames (principal investigator), R Roopun; *Addenbrooke's Hospital, Cambridge*: F Crawley (principal investigator), L Canovas; *Musgrove Park Hospital*, *Taunton*: M Fish (principal investigator), D Sharratt, E Homan, E Greig, C Price, E Fathers.
